# Should organized sport characteristics be considered as a strategy for meeting physical activity guidelines in children?

**DOI:** 10.1177/17579759241237525

**Published:** 2024-03-27

**Authors:** Daniela Rodrigues, Aristides M. Machado-Rodrigues, Augusta Gama, Maria-Raquel G. Silva, Helena Nogueira, Cristina Padez

**Affiliations:** 1 University of Coimbra, CIAS – Research Centre for Anthropology and Health, Portugal; 2 University of Coimbra, DCV – Department of Life Sciences, Portugal; 3 University of Coimbra, Faculty of Sport Sciences and Physical Education, Portugal; 4 Department of Animal Biology, Faculty of Sciences of the University of Lisbon, Portugal; 5 Faculty of Health Sciences, University Fernando Pessoa, Porto, Portugal; 6 Faculty of Arts and Humanities, University of Coimbra, Portugal

**Keywords:** children, organized sports, MVPA, sedentary behaviour, accelerometer

## Abstract

**Background:**

A clearer understanding of the relationships between specific sport context with overall physical activity (PA) and sedentary time (ST) may contribute to the development of more accurate preventive strategies to increase children’s engagement in PA.

**Purpose:**

This study aimed to examine how different organized sports contributed to children’s daily PA and ST.

**Methods:**

PA was measured for seven days via accelerometers, in 410 children aged 6–10 years (49.8% boys). Of those, 332 (53.0% boys) were engaged in an organized sport and were further considered for statistical analyses. Parents reported children’s sport participation (e.g. which sport, number of times per week, duration). The sports were classified into: indoor *vs*. outdoor; individuals *vs*. team; combat *vs*. individual aesthetic *vs*. racing *vs*. invasion. Children’s height and weight were objectively collected. Multiple one-way analyses of covariance were used to examine the effects of sport characteristics on PA and ST. A linear regression, adjusted for children’s sex, age, body mass index and father’s educational level, determined the relationship between being involved in multiple PA and sedentary behaviours with Moderate to Vigorous PA (MVPA) levels.

**Results:**

Although engaged in an organized sport, only 30% of the children achieved the PA recommendations. Sport (compared with active commute and active play) was the best contributor to daily MVPA. Outdoor sports (*vs*. indoor) contributed the most to vigorous PA (VPA) and MVPA. Team sports (*vs*. individual) were significantly associated with lower ST. Children in combat sports accumulated more VPA and MVPA, while those in racing sports showed a higher ST.

**Conclusions:**

Sport participation alone does not guarantee children will reach the PA guidelines, and the type of sport can influence children’s PA levels. Gender-stereotypes in sports may prevent girls from achieving their 60 minutes of MVPA daily.

## Introduction

Physical activity (PA) is associated with several health benefits (1). Current guidelines recommend children accrue 60 min of moderate-to-vigorous physical activity (MVPA) per day (2). However, in Portugal only 30% of children comply with the World Health Organization (WHO) PA guidelines (3). Sedentary time (ST) also increased gradually, which constitutes an additional and independent cardiovascular risk factor (4). Sports contribute between 23% and 60% of children’s daily MVPA (5,6). Organized sport participation also seems more stable over time and has an important role in preventing and reducing childhood obesity (7). Previous studies have shown that most Portuguese children are engaged in an extracurricular sport (>50%) (8,9), but participation rates have declined (3,10) and are constantly lower among females (11).

Sport participation alone does not ensure concordance with PA guidelines, and both physical and sedentary behaviours may co-exist among paediatric lifestyles (12). More time in organized sports may result in reduced time in other activities, such as active play, and some organized sports include sedentary to light intensity activity (e.g. during instruction, or waiting one’s turn) (13,14). Estimates of PA levels (e.g. typically based on metabolic rate) for different activities are available (15). However, most studies have been carried out in adolescents (16) and the amount of MVPA associated with various sports under field conditions is not completely understood. This study aims to examine how participation in organized sports with different characteristics contributes to children’s daily PA and ST.

## Materials and methods

### Participants

The present study is part of the cross-sectional project ObesInCrisis, carried out in Portugal during the winter season of 2016/2017. The sampling design has been described elsewhere (17). Shortly, a total of 118 schools in the cities of Porto, Coimbra and Lisbon were included, with a total of 8472 child-ren (mean age: 7.17 ± 1.91 years, 50.8% male); participation rates were 60%, 58% and 67%, respectively. All the children within those schoo-ls were eligible to participate. For this study, a subsample of 1st-to-4th grade school students from public schools, residing on the Portuguese Midlands was used. Children with missing information on accelerometry were excluded from the sample. Therefore, the sample comprises 410 children aged 6–10 years (8.39 ± 1.18 years). No child had a ch-ronic health condition or disability that could in-fluence their physical movement.

The study, conducted under the principles of the Declaration of Helsinki, was approved by the Portuguese Commission for Data Protection (REF:745/2017) and the Portuguese Ministry of Education (*Direção Geral do Ensino*; REF:0565500003). Written informed consent was obtained from the parents of participating children.

### Instrumentation and procedure

Height and weight were measured by trained researchers at school, in the morning, using a portable stadiometer (SECA^®^, ADE MZ10042, Hamburg, Germany) and an electronic scale (SECA^®^, 813, Hamburg, Germany).

Parents filled in a questionnaire with the question: ‘Does your child participate in any kind of organized sport?’ (yes or no). Organized sport was defined as structured leisure time PA (outside school hours) that is supervised/guided by a coach or a teacher, and involves rules and formal practice. If yes, parents were asked to report the sport(s) practised by the child, the number of sessions per week and the duration (minutes) of each sport session in a typical week. Sports were classified according to: 1) the place where the activity typically takes place (indoor *vs*. outdoor/mix), 2) how the activity is played (individual *vs*. team/mix), and 3) the type of sport (combat, individual aesthetic, including net/court sports because of the sample size, racing, and invasion) as in previous works (18,19). In category 3, when the child practised more than one sport with opposite characteristics (e.g. swimming and football), the classification was made according to the dominant sport (more minutes per week). Those cases were included in ‘outdoor or mix’ and ‘team or mix’ in category 1 and category 2, respectively. Examples are available in the Supplemental material Table S1 online.

Parents answered to 1) ‘How does your child usually get to and from school, and how much time does it take for each travel?’ (Actively: walking or cycling; Passively: motorized vehicles), 2) ‘In his/her free time, about how much time per day is your child usually playing actively (e.g. running and jumping outside, or moving and fitness games inside)?’, 3) ‘In his/her free time, about how much time per day is your child usually engaged in non-screen-based activities, such as reading, puzzles, and dolls?’, and 4) ‘Outside school lessons, how much time does your child usually spend watching TV or using a computer, tablet, video game consoles, or smartphone?’ Possible answers were none, 30 min/day, 60 min/day, 120 min/day, 180 min/day, 240 min/day, and 270 min/day. Father’s educational level was classified as low (⩽9 years), medium (10 to 12 years) or high (university degree).

PA and ST were objectively measured for seven consecutive days. The tri-axial accelerometer, a w*GT3X-BT Actigraph*, was placed over the hip using an elastic belt above the right anterior superior iliac spine; a sample rate of 100 Hz (range 30–100 Hz) was selected. The filtered acceleration signal is digitized and the magnitude is summed over a user-specific period of time (epoch interval) set at 5 s. Participants were instructed to wear the accelerometer during all waking hours except while bathing or doing other water-based activities. Data were downloaded using the *ActiLife 6 software*.

Participants who did not complete a minimum of 600 min/day of accelerometer data after removing sequences of ⩾20 consecutive zero counts were defined as missing data (20,21). Accelerometer output was interpreted using intensity-based cut-points (e.g. sedentary, light, moderate, or vigorous PA). MVPA was calculated by adding the moderate PA (MPA) and the vigorous PA (VPA). Sub-components of PA were expressed in terms of minutes per day and calculated using a specific paediatric cut-point for children (22). Those who accumulated at least 60 min/day in MVPA (weekly average) were classified as meeting PA guidelines (2).

### Data analysis

**Figure 1.**
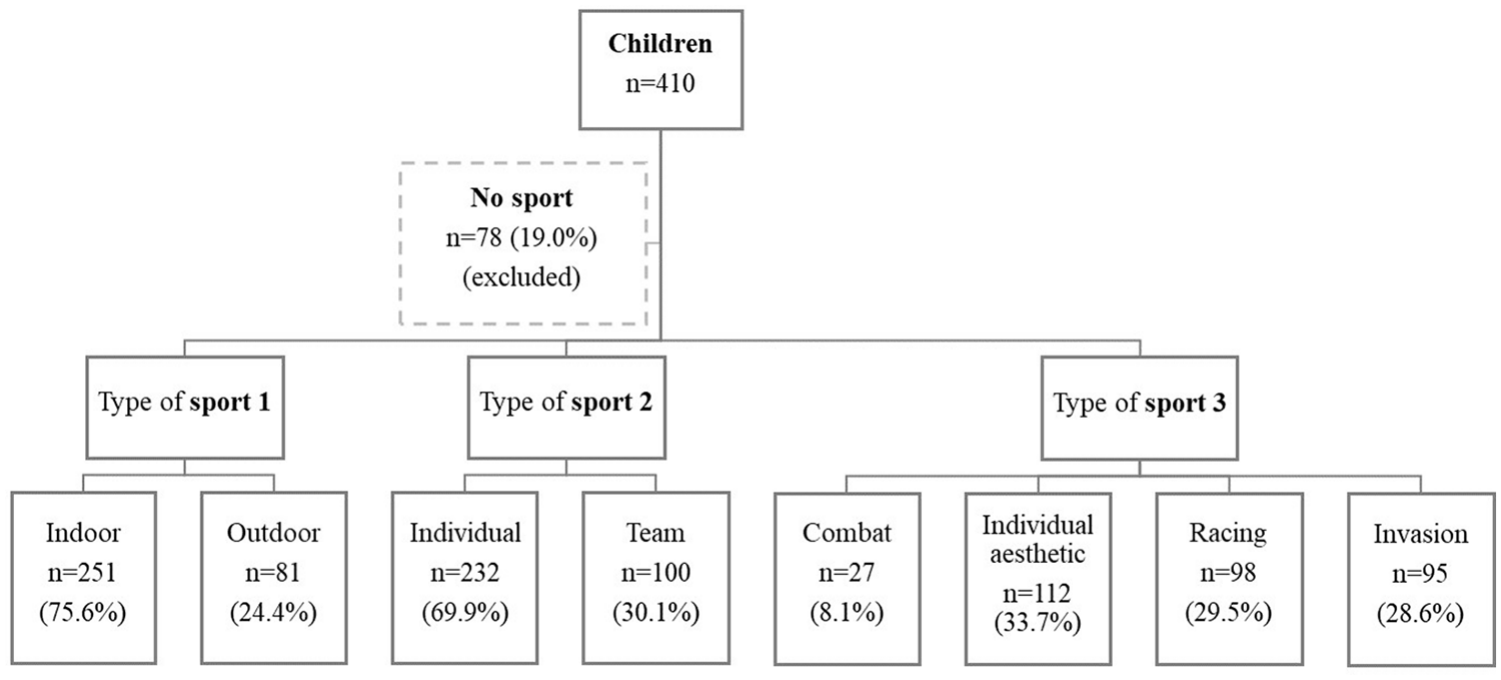
Flow chart of participants who practise and do not practise an organized sport. Examples for indoor sports: basketball and gymnastics; outdoor: football and tennis; individual: dancing and swimming; team: hockey and rugby; combat: judo and karate; individual aesthetic: dancing and yoga; racing: canoe and swimming; invasion: football and water polo. Complete classification of sports according to sports categories is available in Supplemental material Table S1 online.

Of 410 children, 332 were engaged in an extracurricular sport (Figure 1). Supplemental Table S2 shows that children participating in a sport were more frequently male (53.0% *vs*. 47.0% females), had lower BMI (16.8 *vs*. 16.9) and a lower prevalence of overweight (14.8% *vs*. 17.6%) and were mostly from high-educated families (62.4% *vs*. 59.8%). Only children engaged in a sport were considered for further analyses.

Chi-square and Mann–Whitney *U*-test were used to compare differences between sexes in sport participation (categories 1, 2 and 3), BMI, PA guidelines, ST, and PA intensity. To determine the effect of the sport categories (independent variables) in terms of PA intensity (dependent variables), multiple one-way analyses of covariance were run, controlling to children’s sex, age, BMI, school, and father’s education level. Homogeneity of variances (Levene’s test > 0.05) was required in all the analyses. When statistically significant differences were found according to the sport categories, a post hoc Bonferroni test was used to determine specific effects on PA intensity. Linear regression was used to estimate the relationship between engagement (i.e. minutes per day) in sedentary (e.g. indoor play, screen time) and active activities (e.g. commute, sport participation, active play) and achieved daily MVPA. The model was adjusted to all the activities, children’s sex, age, BMI, school, and father education. Significance was set at 5%; data were analysed using IBM SPSS, v.26 SPSS.

## Results

The descriptive data of organized sports participation and characteristics of participants are presented in Table 1. Outdoor (39.2% *vs*. 7.7%) and team sports (51.7% *vs*. 5.8%) were significantly more common in boys than in girls, respectively. Invasion sports were more common among boys (49.4%), while girls were mostly involved in individual aesthetic sports (59.6%). The daily ST was significantly higher in girls than in boys, while the minutes per day in MPA, VPA and MVPA was significantly higher in boys compared with girls. Boys were also significantly more likely to meet the PA guidelines than girls.

**Table 1.** Descriptive characteristics (*n* and %, or mean and standard deviation) of participants engaged in an organized sport in total sample (*N* = 332), boys (*n* = 176) and girls (*n* = 156).

	Total	Boys	Girls	Statistical test
**Sport category 1** ^a^				
Indoor	251 (75.6)	107 (60.8)	114 (92.3)	**χ**^2^ **(1)** **=** **44.52, *p*** **<** **0.001**
Outdoor	81 (24.4)	69 (39.2)	12 (7.7)	
**Sport category 2** ^a^				
Individual	232 (69.9)	85 (48.3)	147 (94.2)	**χ**^2^ **(1)** **=** **82.91, *p*** **<** **0.001**
Team	100 (30.1)	91 (51.7)	9 (5.8)	
**Sport category 3** ^a^				
Combat	27 (8.1)	20 (11.4)	7 (4.5)	**χ**^2^ **(3)** **=** **120.12, *p*** **<** **0.001**
Individual aesthetic	112 (33.7)	19 (10.8)	93 (59.6)	
Racing	98 (29.5)	50 (28.4)	48 (30.8)	
Invasion	95 (28.6)	87 (49.4)	8 (5.1)	
**PA guidelines** ^a^				
Not accomplished	220 (66.3)	101 (57.4)	119 (76.3)	**χ**^2^ **(1)** **=** **13.21, *p*** **<** **0.001**
Accomplished	112 (33.7)	75 (42.6)	37 (23.7)	
**BMI** ^b^	16.81 ± 2.61	16.61 ± 2.04	17.04 ± 3.11	*Z* = –0.60, *p* = 0.55
**Activity on all days (min/day)** ^b^				
Sedentary	506.13 ± 88.29	491.99 ± 83.81	522.08 ± 90.75	***Z* = –2.69, *p* = 0.01**
LPA	180.13 ± 37.22	182.62 ± 37.25	177.32 ± 37.09	*Z* = -0.95, *p* = 0.35
MPA	30.99 ± 9.36	33.29 ± 9.45	28.38 ± 8.56	***Z* = –4.61, *p*** **<** **0.001**
VPA	22.91 ± 10.65	24.39 ± 11.53	21.22 ± 9.32	***Z* = –2.39, *p* = 0.02**
MVPA	53.89 ± 18.91	57.69 ± 19.81	49.60 ± 16.90	***Z* = -3.62, *p*** **<** **0.001**

Bold font denotes statistical significance at the *p* < 0.05 level.

a Tested by chi-square (**χ**^2^ **)**.

b Tested by Mann–Whitney test.

PA: physical activity; BMI: body mass index; LPA: light physical activity; MPA: moderate physical activity; VPA: vigorous physical activity; MVPA: moderate-to-vigorous physical activity

Children practising an outdoor sport accumulated significantly more MPA (~3 min/day), VPA (~4 min/day) and MVPA (~6 min/day) than children in indoor sports (Table 2), while children performing an individual sport accumulated significantly more ST (~41 min/day) than children in team sports (Table 3). There was a statistically significant difference between the type of sport and the daily VPA, MVPA and ST (Table 4). The post hoc test showed that children in combat sports accumulated more VPA than those practising racing sports (~7 min/day). Individual aesthetic sports compared with racing sports also contributed to higher VPA (~4 min/day) and MVPA (~7 min/day). Inversely, racing sports contributed the most to ST (~51 min/day) compared with invasion sports. There were no statistically significant differences between the other groups.

**Table 2.** Physical activity and sedentary levels (minutes per day) of children practising indoor or outdoor sports; one-way analysis of covariance.

Activity		Sport category 1		Statistical test
		Indoor	Outdoor	
Sedentary	*M* (SD)	510.28 (91.27)	495.95 (82.92)	*F*(1,315) = 0.54, *p* = 0.49, partial η2 = 0.00
	*M*_adj_ (SE)	509.02 (5.78)	499.81 (10.66)	
LPA	*M* (SD)	178.35 (37.36)	181.47 (36.46)	*F*(1,315) = 1.11, *p* = 0.37, partial η2 = 0.00
	*M*_adj_ (SE)	177.79 (2.38)	183.22 (4.38)	
MPA	*M* (SD)	29.90 (9.04)	33.43 (9.02)	***F*(1,315) = 5.01, *p* = 0.03, partial η2 = 0.02**
	*M*_adj_ (SE)	**30.09 (0.57)**	**32.86 (1.05)**	
VPA	*M* (SD)	21.95 (10.20)	25.52 (11.40)	***F*(1,315) = 5.98, *p* = 0.02, partial η2 = 0.02**
	*M*_adj_ (SE)	**21.94 (0.68)**	**25.54 (1.25)**	
MVPA	*M* (SD)	51.84 (18.15)	58.95 (19.24)	***F*(1,315) = 6.24, *p* = 0.01, partial η2 = 0.02**
	*M*_adj_ (SE)	**52.03 (1.17)**	**58.40 (2.17)**	

*M*_adj_: = mean adjusted to children’s sex, age, body mass index, school, and father’s educational level; bold font denotes statistical significance at the *p* < 0.05 level.

*M*: mean; SD: standard deviation; SE: standard error; LPA: light physical activity; MPA: moderate physical activity; VPA: vigorous physical activity; MVPA: moderate-to-vigorous physical activity

**Table 3.** Physical activity and sedentary levels (minutes per day) of children practising individual or team sports; one-way analysis of covariance.

Activity		Sport category 2		Statistical test
		Individual	Team	
Sedentary	*M* (SD)	519.02 (87.53)	477.04 (87.27)	***F*(1,315) = 11.01, *p* = 0.00, partial η2 = 0.03**
	*M*_adj_ (SE)	**518.72 (6.01)**	**477.76 (9.98)**	
LPA	*M* (SD)	180.79 (35.89)	175.06 (39.82)	*F*(1,315) = 1.87, *p* = 0.14, partial η2 = 0.01
	*M*_adj_ (SE)	181.18 (2.51)	174.13 (4.16)	
MPA	*M* (SD)	29.94 (8.87)	32.76 (9.55)	*F*(1,315) = 0.76, *p* = 0.42, partial η2 = 0.00
	*M*_adj_ (SE)	30.45 (0.61)	31.53 (1.01)	
VPA	*M* (SD)	22.13 (10.13)	24.49 (11.56)	*F*(1,315) = 1.08, *p* = 0.31, partial η2 = 0.00
	*M*_adj_ (SE)	22.37 (0.72)	23.92 (1.20)	
MVPA	*M* (SD)	52.08 (17.89)	57.25 (20.00)	*F*(1,315) = 1.04, *p* = 0.33, partial η2 = 0.00
	*M*_adj_ (SE)	52.82 (1.25)	55.45 (2.08)	

*M*_adj_: = mean adjusted to children’s sex, age, body mass index, school, and father’s educational level; bold font denotes statistical significance at the *p* < 0.05 level.

*M*: mean; SD: standard deviation; SE: standard error; LPA: light physical activity; MPA: moderate physical activity; VPA: vigorous physical activity; MVPA: moderate-to-vigorous physical activity

**Table 4.** Physical activity and sedentary levels (minutes per day) of children by sport categories (e.g. combat, individual aesthetic, racing or invasion sports); one-way analysis of covariance.

Activity		Sport category 3				Statistical test
		Combat	Individual aesthetic	Racing	Invasion	
Sedentary	*M* (SD)	502.42 (95.75)	516.27 (91.72)	526.46 (81.40)	475.61 (85.52)	***F*(3,313) = 4.68, *p* = 0.00, partial η2 = 0.04**
	*M*_adj_ (SE)	**508.78 (17.31)**	**508.36 (9.22)**	**530.56 (9.01)**	**479.35 (10.26)**	
LPA	*M* (SD)	181.46 (29.41)	179.00 (37.26)	181.91 (35.52)	175.67 (40.67)	*F*(3,313) = 0.82, *p* = 0.46, partial η2 = 0.01
	*M*_adj_ (SE)	175.58 (7.25)	183.21 (3.86)	180.35 (3.77)	173.76 (4.30)	
MPA	*M* (SD)	32.21 (9.95)	29.94 (8.76)	29.33 (8.46)	32.88 (9.76)	*F*(3,313) = 2.15, *p* = 0.09, partial η2 = 0.02
	*M*_adj_ (SE)	30.88 (1.74)	31.95 (0.93)	28.88 (0.91)	31.23 (1.03)	
VPA	*M* (SD)	26.72 (11.91)	22.74 (9.73)	20.08 (9.43)	24.66 (11.79)	***F*(3,313) = 4.70, *p* = 0.00, partial η2 = 0.04**
	*M*_adj_ (SE)	**26.15 (2.05)**	**24.00 (1.09)**	**19.58 (1.07)**	**23.78 (1.21)**	
MVPA	*M* (SD)	58.93 (21.29)	52.68 (17.58)	49.41 (16.41)	57.54 (20.42)	***F*(3,313) = 3.66, *p* = 0.01, partial η2 = 0.03**
	*M*_adj_ (SE)	**57.04 (3.56)**	**55.95 (1.90)**	**48.46 (1.86)**	**55.01 (2.11)**	

*M*_adj_: = mean adjusted to children’s sex, age, body mass index, school, and father’s educational level; bold font denotes statistical significance at the *p* < 0.05 level.

*M*: mean; SD: standard deviation; SE: standard error; LPA: light physical activity; MPA: moderate physical activity; VPA: vigorous physical activity; MVPA: moderate-to-vigorous physical activity

A linear regression was used to test whether time in different activities significantly predicted the MVPA (Table 5). The overall regression was sta-tistically significant and the model explained 12% of the variance in children’s MVPA. After adjustment, children accumulated more minutes per day in MVPA if they spent more time in active play and in extracurricular sports; however, the coefficients’ units were small (β = 0.03 and β = 0.20, respectively).

**Table 5.** Linear regression representing the relationship between time in physical and sedentary activities and children’s daily moderate-to-vigorous physical activity.

	β	*t*	*p*-value
Active commute	0.02	0.26	0.80
Active play	**0.12**	**1.90**	**0.05**
Screen media use	–0.10	–1.67	0.10
Play indoor	–0.09	–1.35	0.18
Extracurricular sport	**0.20**	**3.25**	**0.00**
*R*	0.35		
*F*(10,259)	**3.62**		**<0.001**

Model is adjusted to all the variables plus children’s sex, age, body mass index, school, and father education level; bold font denotes statistical significance at the *p* < 0.05 level.

## Discussion

Only one third of children achieved the recommen-ded 60 min/day of MVPA, even though they were engaged in organized sports. This is somewhat in line with previous studies, suggesting values between 20% and 30%, including in Portugal (3,23,24). The results highlight the need for more (and effective) strategies to achieve the global target of a 15% reduction in insufficient PA by 2030 (25).

A positive association between sport participation and accelerometer measured PA has been previously reported (26–29). We found that children who were practicing a sport (versus those not practicing any sport) accumulated ~4 min/day more of MVPA; the effect was greater in girls than in boys (although not statistically significant in any sex). Most children obtain PA from more than one context. Children are likely to engage in unorganized physical activities, such as active commute and physical education classes, since these are less dependent on resources and parental involvement. It may be that children engaged in more unorganized PA will have less time and desire to practise an organized sport, explaini-ng the similar MVPA values found in this study. Nevertheless, a higher MVPA level potentially resulting from participation in organized sports, even if small, can further benefit a wide range of health indicators in children, such as bone strength, motor development, fitness and psychosocial health (1).

Team and outdoor sports were the best contributors to children’s PA, which is in line with previous studies (13,26,27,30,31). Racing sports contributed the most to children’s daily ST, while combat and invasion sports were associated with greater VPA and MVPA. This is probably related to the class content (e.g. knowledge, management, game play and fitness) and the number of sessions per week. Most children practising racing sports, mostly swimming in our sample, had a single sport session (~60 min/week; data not shown), which is insufficient to increase overall weekly health-related PA levels (25). Swimming is a common sport in Portugal (32), including in the present sample, in which 27% of boys and 31% of girls were practising it (data not shown). However, if considering the duration per week, football (e.g. invasion sport) was the most played sport by boys, while gymnastics (e.g. individual aesthetic sport) was favoured by girls. Moreover, the accelerometer had to be removed during aquatic activities, which is a major limitation of the study. In fact, previous studies have suggested that swimming is a viable option to provide children with ample MVPA (33). For this reason, present findings should be interpreted with caution.

Boys seem to prefer MVPA in the form of team sports in which strength and competitiveness pre-dominate, while girls often engage in MPA such as dancing or skating, or activities related to body shape and health with a more aesthetic orientation (34). A phenomenon of gendered sports seems evident, whereby certain sports may be seen as masculine or feminine (35). Sports segregation according to gender roles may be related to the fear of being judged or bullied if gender norms are not conformed to (Martínez-Andrés *et al*. (36)). Also, a previous study suggested an environmental mismatch in Portugal that undermines girls’ opportunities to play sports (37).

Further research to understand why children choose to participate in certain sports is needed. Different activities may enhance different cognitive, social and motor skills, and high-demand sports, such as football, have shown to combine those abilities (38). Team sports seem to be more sup-portive in terms of strategy to fulfil PA recom-mendations, so motivating children to engage in team sports has the potential for improvement in terms of the total amount of daily PA. This may be particularly important for girls, since less than 24% of them were achieving the MVPA guidelines (*vs*. ~43% of boys), which is consistent with previous research showing that girls are often less active than boys, independently of age (11,39,40).

### Implications for policy, practice, and equity

PA encompasses exercise, sports and physical activities performed as part of daily living, occ-upation, leisure or active transportation. The Global Action Plan on PA promotes all of those activities in children (plus reduction in screen time), considering that all movements count. In addition to personal and social development, sports may provide significantly more VPA than activities of daily living (e.g. transportation), which seems to be needed in order to achieve healthier body fat profiles (41).

Sports typically are not free. School-based pro-grammes are a key component to increase sport participation and PA in children, particularly those from socioeconomically disadvantaged families. School programmes should accommodate children’s preferences, since one of the most dominant factors related to sport dropout is participants’ lack of enjoyment/fun (42). Simultaneously, physical edu-cation classes should be adapted to encourage children to try new sports (which may break sport-related gender stereotypes), as well as develop positive attitudes and behavioural skills. Media representation of female sport is another area of importance that can be intervened.

### Limitations

PA was objectively measured. Sport participati-on data were collected using a standardized qu-estionnaire, with little risk of recall bias (sports were being practised). Despite a large enough sample to provide valid information, it is not possible to assert its representativeness. The analysis was adjusted for children’s sex, age, BMI, school, and father’s education, potential sources of confounding of PA levels and sport participation. However, the cross-sectional nature precludes any inference about causality, and we cannot rule out that results may be explained by unmeasured reverse causality or confounders. For instance, no factors affecting sp-orts involvement and performance were collected (e.g. motor competence, sport preferences or reason to practise a specific sport). Underestimation of PA may have occurred because of 1) inherent limitations of the accelerometry to detect some types of PA (e.g. cycling) and to capture aquatic PA (e.g. swimming), and 2) the data collection during the winter season (e.g. decreased outdoor time).

## Conclusions

Attaining the recommended levels of PA is a central aspect of health and wellbeing. An essential step in PA promotion is to identify and measure its suboptimal behaviours. Present findings suggest that sport participation may be a viable strategy to increase overall health-related PA levels. However, sport participation alone does not guarantee that children will reach the WHO PA guidelines. The manner and context in which a sport is delivered can dramatically influence the PA amount and intensity of participants. It seems necessary to break down gender stereotypical sporting behaviour to foster a diverse sport environment to both boys and girls and thereby contribute to their health.

## Supplemental Material

sj-docx-1-ped-10.1177_17579759241237525 – Supplemental material for Should organized sport characteristics be considered as a strategy for meeting physical activity guidelines in children? Supplemental material, sj-docx-1-ped-10.1177_17579759241237525 for Should organized sport characteristics be considered as a strategy for meeting physical activity guidelines in children? by Daniela Rodrigues, Aristides M. Machado-Rodrigues, Augusta Gama, Maria-Raquel G. Silva, Helena Nogueira and Cristina Padez in Global Health Promotion

sj-docx-2-ped-10.1177_17579759241237525 – Supplemental material for Should organized sport characteristics be considered as a strategy for meeting physical activity guidelines in children? Supplemental material, sj-docx-2-ped-10.1177_17579759241237525 for Should organized sport characteristics be considered as a strategy for meeting physical activity guidelines in children? by Daniela Rodrigues, Aristides M. Machado-Rodrigues, Augusta Gama, Maria-Raquel G. Silva, Helena Nogueira and Cristina Padez in Global Health Promotion
