# Supplementary material for: Should organized sport characteristics be considered as a strategy for meeting physical activity guidelines in children?
Source: Glob Health Promot. 2024 Mar 27;31(4):75–84. doi: 10.1177/17579759241237525 (PMC11636020; doi:10.1177/17579759241237525)
Supplement: sj-docx-1-ped-10.1177_17579759241237525 – Supplemental material for Should organized sport characteristics be considered as a strategy for meeting physical activity guidelines in children? [file sj-docx-1-ped-10.1177_17579759241237525.docx]

**Table S1.** Classification of sports according to sports categories.

| **Organized sports practiced by the children** | Aikido, Athletics, Basketball, Canoe/Kayak, Dancing, Figure skating, Football, Futsal, Gymnastics, Hockey, Horse riding, Judo, Karate, Kickboxing, Krav maga, Rugby, Surf, Swimming, Table tennis, Tennis, Volleyball, Water polo, Yoga | |
| --- | --- | --- |
|  | |  |
| **Category 1** |  | **N** |
| Indoor | Aikido, Basketball, Dancing, Figure skating, Futsal, Gymnastics, Hockey, Judo, Karate, Kickboxing, Krav maga, Swimming, Table tennis, Volleyball, Water polo, Yoga | 251 |
| Outdoor | Athletics, Canoe/Kayak, Football, Horse riding, Rugby, Surf, Tennis | 81 |
| **Category 2** |  | **N** |
| Individual | Aikido, Athletics, Canoe/Kayak, Dancing, Figure skating, Gymnastics, Horse riding, Judo, Karate, Kickboxing, Krav maga, Surf, Swimming, Table tennis, Tennis, Yoga | 232 |
| Team | Basketball, Football, Futsal, Hockey, Rugby, Volleyball, Water polo | 100 |
| **Category 3†** |  | **N** |
| Combat | Aikido, Judo, Karate, Kickboxing, Krav maga | 27 |
| Individual aesthetic | Dancing, Figure skating, Gymnastics, Horse riding, Surf, Yoga | 104 |
| Individual aiming | None | 0 |
| Racing | Athletics, Canoe/Kayak, Swimming | 98 |
| Net/Court | Table tennis, Tennis, Volleyball | 8 |
| Invasion | Basketball, Football, Futsal, Hockey, Rugby, Water polo | 95 |
| Fielding | None | 0 |
| Target | None | 0 |

*Note*. †Classification according to Livingston and Forbes (2016) and Stefani (1999).
