# Supplementary material for: Should organized sport characteristics be considered as a strategy for meeting physical activity guidelines in children?
Source: Glob Health Promot. 2024 Mar 27;31(4):75–84. doi: 10.1177/17579759241237525 (PMC11636020; doi:10.1177/17579759241237525)
Supplement: sj-docx-2-ped-10.1177_17579759241237525 – Supplemental material for Should organized sport characteristics be considered as a strategy for meeting physical activity guidelines in children? [file sj-docx-2-ped-10.1177_17579759241237525.docx]

**Tabela S2.** Descriptive characteristics of participants.

|  | **All children (n=410)** | **Children participating in a sport (n=332)** | **Statistical Test⸸** |
| --- | --- | --- | --- |
| Sex (n, %)**†** |  |  | **χ^2^(1)=7.40, p=0.01** |
| Male | 204 (49.8) | 176 (53.0) |  |
| Female | 206 (50.2) | 156 (47.0) |  |
| Age (years) (mean, SD)**‡** | 8.3 (1.2) | 8.3 (1.1) | Z=-0.28, p=0.78 |
| BMI (kg/m^2^) (mean, SD)**‡** | 16.9 (2.6) | 16.8 (2.0) | **Z=-2.24, p=0.03** |
| IOTF (n, %)**†** | | | **χ^2^(1)=9.46, p=0.00** |
| Normal | 338 (82.4) | 283 (85.2) |  |
| Overweight + obesity | 72 (17.6) | 49 (14.8) |  |
| Activity on all days (min, SD)**‡** | | |  |
| Sedentary | 506.9 (96.9) | 506.1 (88.2) | Z=-0.08, p=0.94 |
| LPA | 178.9 (37.7) | 180.1 (37.2) | Z=-1.06, p=0.29 |
| MPA | 30.7 (9.3) | 30.9 (9.3) | Z=-1.10, p=0.27 |
| VPA | 22.4 (10.2) | 22.9 (10.6) | Z=-1.45, p=0.15 |
| MVPA | 53.1 (18.4) | 53.8 (18.9) | Z=-1.26, p=0.21 |
| PA guidelines (n, %)**†** | | | χ^2^(1)=0.25, p=0.62 |
| Not accomplished | 136 (33.2) | 112 (33.7) |  |
| Accomplished | 136 (33.2) | 112 (33.7) |  |
| Father education level (n, %)**†** | | | **χ^2^(2)=15.60, p<0.001** |
| Low | 36 (9.2) | 21 (6.5) |  |
| Medium | 122 (31.0) | 100 (31.1) |  |
| High | 235 (59.8) | 201 (62.4) |  |

*Note*. LPA, light physical activity; MPA, moderate physical activity; MVPA, moderate to vigorous physical activity; **⸸** Grouping variable is “does not do sport” and “does do sport”; † tested by chi-square; ‡ tested by Mann-Whitney test; bold font denotes statistical significance at the p<0.05 level.
